# miRNA-200c-3p deficiency promotes epithelial-mesenchymal transition in triple-negative breast cancer by activating CRKL expression

**DOI:** 10.1007/s12672-024-01004-1

**Published:** 2024-05-08

**Authors:** Fangfang Nie, Qinfang Zhang, WeiNa Ma, Jun Yan

**Affiliations:** 1 Department of Oncology, Jiading District Central Hospital Affiliated Shanghai University of Medicine & Health Sciences, No. 1 Chengbei Road, Jiading District, Shanghai, 201800 China; 2 Department of Pharmacy, Jiading District Central Hospital Affiliated Shanghai University of Medicine & Health Sciences, Shanghai, 201800 China

**Keywords:** Malignant phenotype, Epithelial-mesenchymal transition, Triple-negative breast cancer, CRKL

## Abstract

Epithelial-mesenchymal transition (EMT) plays an important role in malignant progression of Triple-negative breast cancer (TNBC). Many studies have confirmed that miRNA-200c-3p is related to EMT. And we found that it is involved in the regulation of EMT, but the exact mechanism is unclear. CRKL is highly expressed in a variety of tumors and plays a role in EMT. In this study, the potential targets of miRNA-200c-3p were searched in miRPathDB, Targetscan and PicTar. And there are 68 potential targets at the intersection of the three databases. Then, bioinformatics and text mining performed by Coremine Medica, and found that among 68 potential targets, CRKL has the strongest correlation with EMT in TNBC. Therefore, we speculated that miRNA-200c-3p involvement in EMT might be related to CRKL. To verify miRNA-200c-3p inhibits the malignant phenotype of TNBC by regulating CRKL, RT‒PCR, western blotting, Clonal formation assays,CCK-8 proliferation assays, transwell invasion assays, Luciferase reporter assay and nude mouse transplantation tumor assay were performed. In this study, we found that miRNA-200c-3p is under-expressed and EMT-related genes are up-regulated in TNBC, and miRNA-200c-3p can inhibit cancer cell proliferation, invasion and the expression of EMT-related genes and proteins in TNBC. Further research confirmed that miRNA-200c-3p could inhibit EMT by inhibiting the expression of CRKL that directly combining CRKL gene.

## Introduction

Triple-negative breast cancer (TNBC) refers to breast cancer that is negative for estrogen receptor (ER), progesterone receptor (PR) and proto-oncogene HER-2 by immunohistochemical examination of cancer tissue. This type of accounts for 10.0–15% of all pathological types of breast cancer [1, 2]. It has special biological behavior and clinicopathological characteristics, and the prognosis is worse than other types. Typically exhibits aggressive behavior, including earlier recurrence, metastasis, high mortality and frequently chemotherapy resistance. Although conventional treatments like surgery and radiotherapy have achieved considerable progress in clinic, the 5-year survival rate of TNBC patients remains lower than 15% [3, 4]. And TNBC has an extremely malignant phenotype. And the clinical treatment effect is very poor at present, Therefore, both basic and clinical studies are necessary for TNBC. More and more studies have displayed that EMT- related mechanisms play an important role in the malignant progression of TNBC.

MicroRNAs(miRNAs) are a class of endogenous small RNAs with a length of about 20 to 24 nucleotides which play a negative regulation role[5].They regulate the expression of target genes by means of the degradation of target gene mRNA or post-transcriptional inhibition of protein translation. MiRNAs are closely involved in cell proliferation, differentiation, programmed cell death, migration and drug resistance. In the same time, their abnormal expression plays a role in cancer inhibition or cancer promotion in a variety of cancer [6–9]. At present, miRNA-200c-3p has been studied in a variety of tumors, and many studies have displayed that miRNA-200c-3p is related to EMT [10–12]. However, the mechanism of miRNA-200c-3p regulation of EMT in TNBC has not been fully elucidated [13–16]. CRKL (CRK Like Proto-Oncogene) as a number of the Crk family that encodes a protein kinase that contains the SH2 and SH3 (SRC homology) domains, which is expressed in many cancer tissues [17, 18]. Recently, CRKL has also been reported to be closely related to EMT of tumors [19–21]. However, the relationship between the CRKL and the miRNA-200c-3p in TNBC still need to be confirmed. This study compared the differential expression levels of the miRNA-200c-3p and the CRKL in triple negative breast cancer tissues and adjacent tissues. And we also investigated the functional roles of miRNA-200c-3p and the CRKL in TNBCs.In the same time, we confirmed that the CRKL maybe a direct target of miRNA-200c-3p. Hence, miRNA-200c-3p and CRKL may become potential therapeutic targets for TNBC.

## Materials and methods

### Tissue samples

This work is approved by The Ethics Committee of Jiading District Central Hospital Affiliated Shanghai University of Medicine &Health Sciences. All methods were performed in accordance with the relevant guidelines and regulations.8 pairs of TNBC and their matched adjacent tissue specimens were collected from our hospital. All specimens were confirmed by pathology then stored at − 80 °C. None of the patients received any other treatment before surgery such as preoperative radiotherapy or chemotherapy. In the same time, they all signed informed consent.

### Cell culture

Cell line (MCF-10A, MDA-MB-231, MDA-MB-468, BT20) were obtained from Institute of Chinese Academy of Sciences. Cells were cultured in DMEM (Gibco, USA) supplemented with 10% fetal bovine serum (FBS) (Gibco, USA) and anti-biotics (Sigma, USA) in a humidified chamber at 37 ℃ with 5% CO_2_.

### Reagents

Micro-RNA, control mimics, miRNA-200c-3p mimics and miRNA-200c-3p inhibitor were purchased from Han heng Science and Technology (Shanghai) Co., LTD. Cholesterol-conjugated miRNAs (Chol-agomiR-NC and CholagomiR-200c-3p) were purchased from Gene Pharma (Shanghai, China). The specificity of miRNA-200c-3p mimic and miRNA-200c-3p inhibitor were confirmed by several recent publications [22].

### Cell proliferation assay

The cells viability assay was executed with the Cell Counting Kit-8 (CCK-8) assay (Dojindo, Kumamoto, Japan). In short, cells were seeded in 96-well plates and incubator for 24 h. The CCK-8 reagent (10 μL) was added to each well, then cells were incubated at 37 °C for 1 h. Absorbance was measured at 450 nm using an Multiskan Spectrum reader (Thermo Scientific, USA).

### Cell migration and invasion assay

Transwell (Corning, MA) was used to analyze cell migration as previously described [23]. Briefly, the undersurface of the upper chamber was coated with 10 μg/mL of collagen I. Cells were suspended in a density of 10^6^ cells/mL and 100 μL of cell suspension was added into the upper chamber of each well. Cells were allowed to migrate for 24 h, cells on the undersurface of the upper chamber were then stained and counted under a phase contrast microscope.

### Wound healing assay

When the cells confluence reached about 80–90%, the 200 μL sterile pipette tip was used for scratching the layers. Then, the scratched cells were washed away by phosphate buffer saline (PBS; Vicmed, China). 2% FBS was added into the fresh medium. The culture dishes were placed into the incubator in the same environment as the cell culture. After 24 h incubation, the scratches were captured with a microscope. MShot Image Analysis System was applied for images.

### Colony formation

Cells were constructed to the single cell suspension for counting, with 500 cells in 60 mm culture medium. After 12–16 days culture process, the cells were washed 3 times with PBS, then fixed with paraformaldehyde for 15 min, and finally stained with crystal violet for 15 min. Number of clones with > 50 cells was determined under microscope (Olympus Corp., Tokyo, Japan).

### Real-time polymerase chain reaction analysis

Total RNA was extracted from cells with Trizol reagent (Takara Bio, Japan), following the manufacture’s protocols. The quantity of extracted RNA was determined using the Nanodrop 2000 spectrophotometer (NanoDrop Products, Wilmington, Del.). RNA was reverse transcribed to cDNA using a Prime Script RT reagent Kit (Takara). The mRNA expression was normalized to that of β-actin. Polymerase chain reaction amplification was performed using SYBR Premix Ex Taq (Takara). Amplification and qRT-PCR measurements were carried out using the Applied Biosystems 7500 Fast Real-Time PCR System (Applied Biosystems, Foster City, Calif.). The average of genes was normalized to β-actin as an endogenous housekeeping gene.

### Western blot assay

Equivalent amounts of protein (30 μg per lane) were loaded and separated by 8%-12% SDS-PAGE gels, and transferred PVDF membrane. After the transfer was completed, the membrane was blocked 30 min and then incubated overnight at 4° with specific primary antibodies as follows: antibodies against E-Cadherin, Cytokeratin, Vinmentin, N-Cadherin and CRKL were obtained from Abcam, β-actin was purchased from Biyuntian Biotechnology Co., LTD. After that the membranes were washed and incubated with secondary antibody for 1 h at room temperature. The bands of proteins were visualized using the ECL Plus system (GE Healthcare, Little Chalfont, United Kingdom). Image J (Scion Corporation) was used to quantify the optical density of each band.

### Luciferase reporter assay

Wild type (WT) or mutant (MT) 3′-UTR of CRKL was amplified and cloned into pmiR-RB-REPORTTM Luciferase vector. Then 3,-UTR luciferase activity was measured using a Dual-Luciferase1 Reporter Assay Kit (Promega, Madison, USA). 3′-UTR of CRKL and corresponding miRNA vectors were co-transfected into cells, respectively. After co-transfection 48 h, the luciferase activity was measured using a Dual-Luciferase1 Reporter Assay Kit (Promega, Madison, USA).

### Nude mouse transplantation tumor assay

Nude mice (Shanghai Slack Laboratory Animal CO.,LTD) were injected subcutaneously with 5*10^6^cells/200 μL (MDA-MB-231). Tumor sizes were measured every 4 days after subcutaneous injection. After 1 weeks, total of 1 OD Chol-agomiR-NC or Chol-agomiR-200c-3p was injected at 3 different spots on each established tumor. The mice were sacrificed by cervical dislocation under anesthesia, and tumors were removed for the volumes measurement after 3–4 weeks.

### Statistical analysis

Data were presented as Mean ± SD and analyzed by GraphPad Prism 6 software (GraphPad Software, USA). The t-test and one-way ANOVA were employed to analyze continuous variable. The value of P < 0.05 was considered to have statistical significance. All experiments were performed at least thrice.

## Results

### miRNA-200c-3p is under-expressed and EMT-related genes are up-regulated in TNBC

**Fig. 1:**
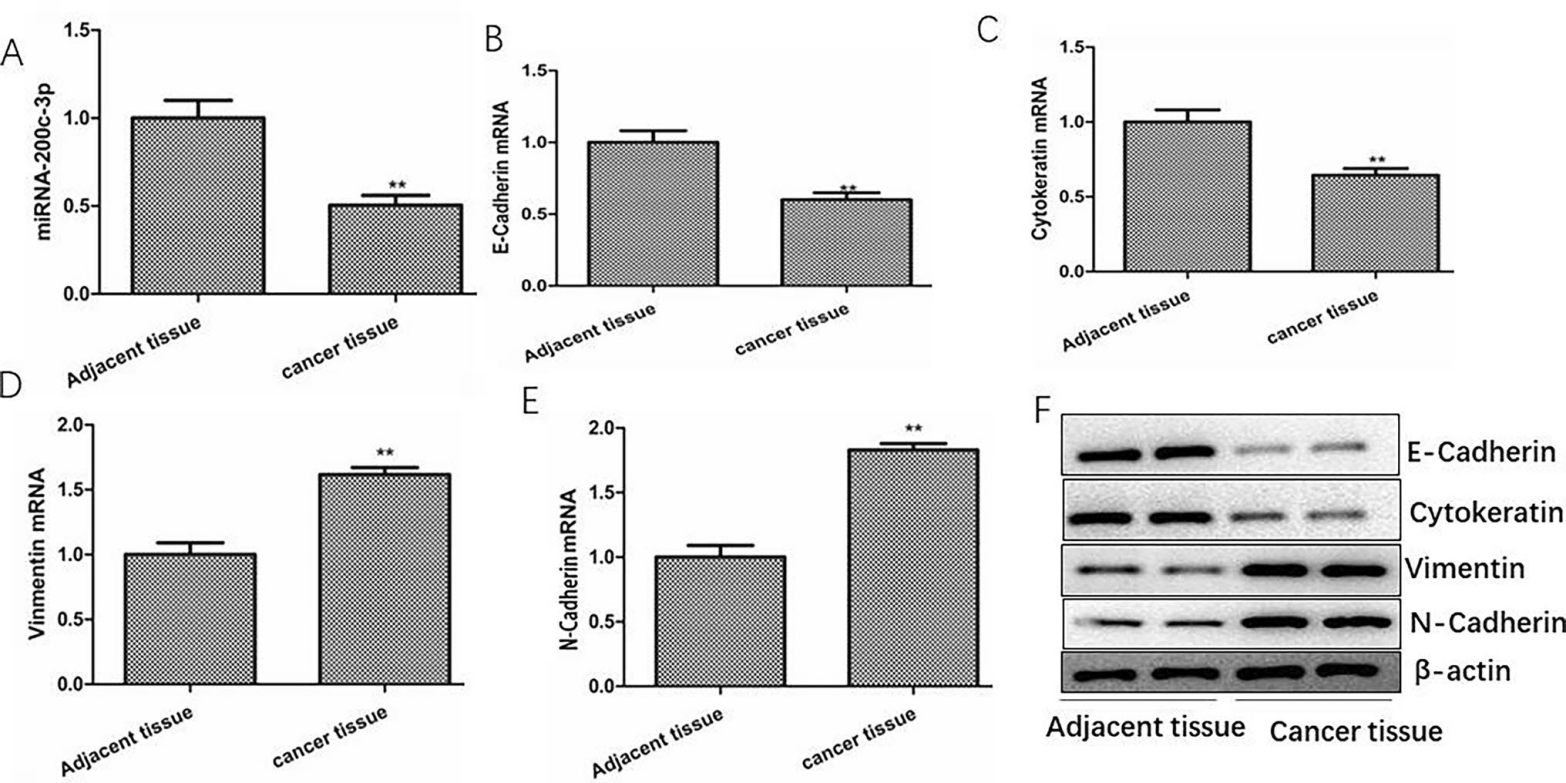
The expression of miRNA-200c-3p, EMT-related genes and proteins in cancer and matched tumor-adjacent tissues. **A** The expression of miRNA-200c-3p in cancer tissues. **B** The expression of E-cadherin mRNA in TNBC tissues detected by RT-PCR. **C** The expression of Cytokeratin mRNA in TNBC tissues detected by RT-PCR. **D** The expression of Vimentin mRNA in TNBC tissues detected by RT-PCR. **E** The expression of N-cadherin mRNA in TNBC tissues detected by RT-PCR. **F** The expression of E-cadherin, Cytokeratin, Vimentin, N-cadherin proteins in TNBC tissues detected by western blotting. The values are presented as the means (SD), **p < 0.01 versus control group. All experiments were repeated three times

RT-PCR was performed to measure the expressions of miRNA-200c-3p in TNBC and matched tumor-adjacent tissues. Results indicated that the levels of miRNA-200c-3p in TNBC tissues were lower than those in matched adjacent tissues (P < 0.01, Fig. 1A). In TNBC, Vimentin, N-Cadherin mRNA levels were highly expressed and E-Cadherin, Cytokeratin mRNA levels were down-regulated (Fig. 1B–E), As the same time, the results of western blotting were consistent with those of RT-PCR (Fig. 1F), which indicated that EMT is enhanced in TNBC.

### miRNA-200c-3p can inhibit cancer cell proliferation and invasion

**Fig. 2:**
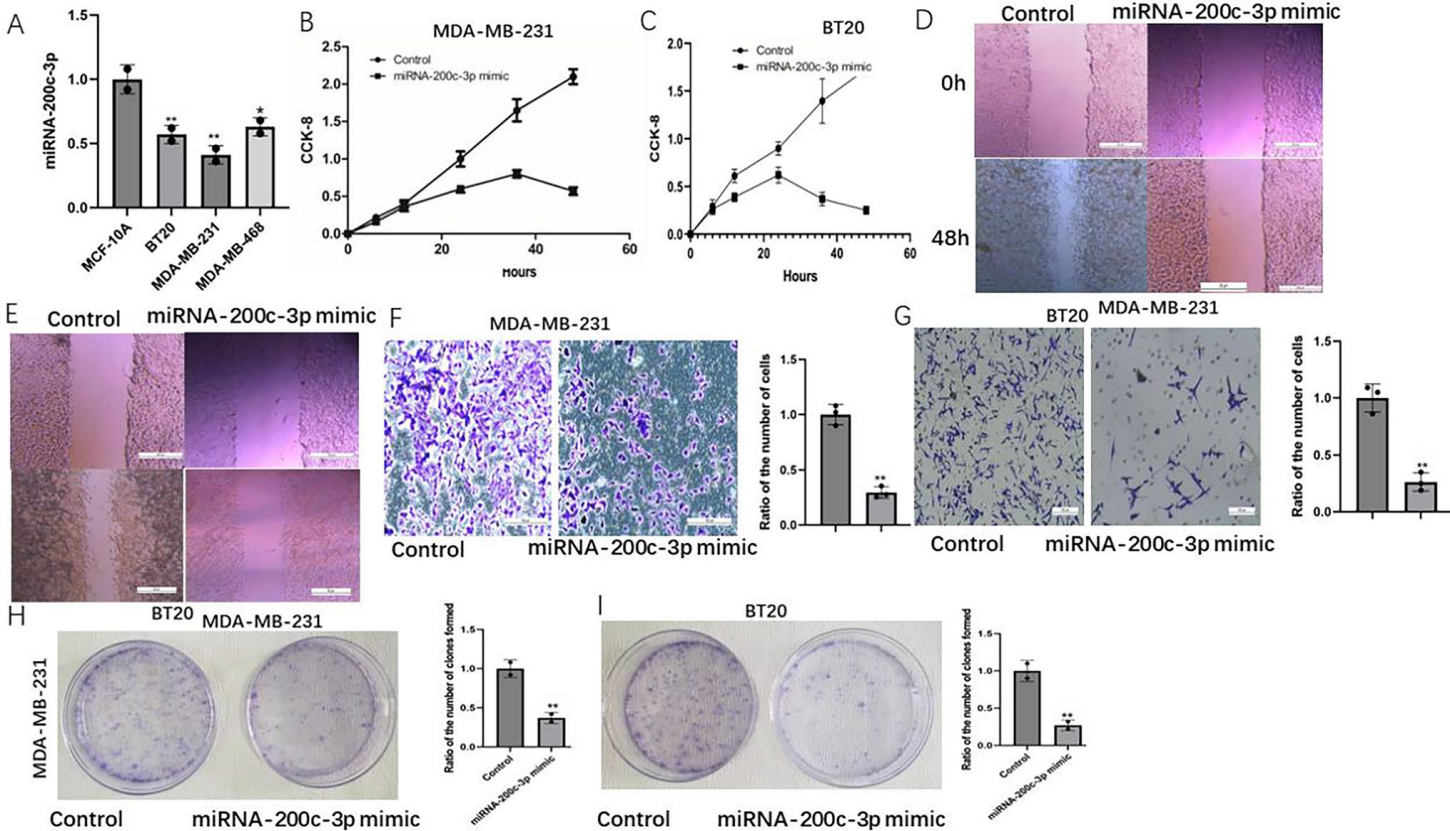
Effects of miRNA-200c-3p on TNBC cells proliferation and invasion. **A** miRNA-200c-3p mRNA level in normal breast cell lines and TNBC lines. **B** CCK-8 to verify the effect of miRNA-200c-3p on TNBCs (MDA-MB-231) proliferation. **C** CCK-8 to verify the effect of miRNA-200c-3p on TNBCs (BT20) proliferation. **D** Cell wound scratch assay to verify the effect of miRNA-200c-3p on TNBCs (MDA-MB-231) migration. **E** Cell wound scratch assay to verify the effect of miRNA-200c-3p on TNBCs (BT20) migration. **F** Transwell assay to verify the effect of miRNA-200c-3p on TNBCs (MDA-MB-231) invasion. **G** Transwell assay to verify the effect of miRNA-200c-3p on TNBCs (BT20) invasion. **H** Colony formation in different groups (MDA-MB-231). I. Colony formation in different groups (BT20). The values are presented as the means (SD), **p < 0.01 versus control group. All experiments were repeated three times

miRNA-200c-3p mRNA level in normal breast cell lines and TNBC lines were performed by RT-PCR, and we found that miRNA-200c-3p decreased in TNBC lines. It is more obvious in MDA-MB-231 and BT20 (Fig. 2A), so we used BT20 and MDA-MB-231 for our follow-up experiments. To determine how miRNA-200c-3p effected TNBC cell growth, invasiveness and migration capability, we performed CCK-8, clone formation assay, cell wound scratch assay and transwell assay on TNBC cells. In a growth period of 48 h, miRNA-200c-3p-mimic inhibited cell growth obviously compared to control group (Fig. 2B, C). Cell wound scratch assay showed that miRNA-200c-3p mimic could be significantly inhibited the migration of tumor cells (Fig. 2D, E). Treatment of miRNA-200c-3p mimic resulted in invasion inhibition in TNBC lines compared to control group (Fig. 2F, G). Clone formation assay showed that miRNA-200c-3p mimic could be significantly inhibited clone formation assay tumor cells (Fig. 2H, I). These results demonstrate that miRNA-200c-3p specifically inhibits TNBC cell proliferation and invasion.

### miRNA-200c-3p can inhibit the expression of EMT-related genes and proteins in TNBC

**Fig. 3:**
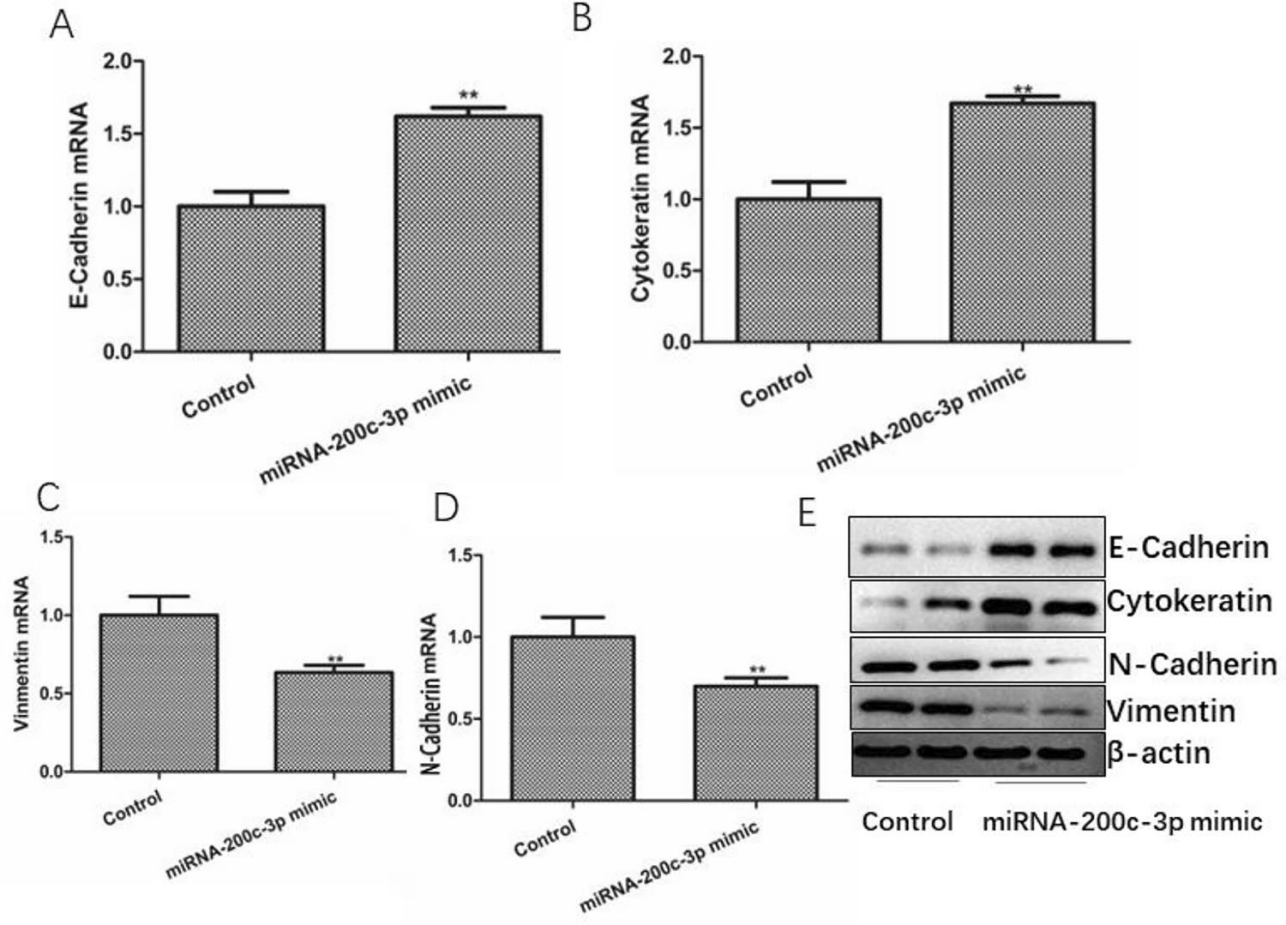
Effects of miRNA-200c-3p on TNBC cells EMT. **A** The expression of E-Cadherin mRNA level. **B** The expression of Cytokeratin mRNA level. **C** The expression of Vimentin mRNA level. **D** The expression of N-Cadherin mRNA level. E. The protein expression of E-Cadherin, Cytokeratin, Vimentin, N-Cadherin and β-actin. The values are presented as the means (SD), **p < 0.01 versus control group. All experiments were repeated three times

To investigate whether miRNA-200c-3p effect the expression of EMT-related genes and proteins of EMT in TNBC, western blotting and RT-PCR were performed to measure the expressions of EMT-related genes and proteins. We found that miRNA-200c-3p-mimic increased the expression of E-cadherin, cytokeratin (Fig. 3A, B) and decreased the expression of vimentin and N-cadherin (Fig. 3C, D). The results of western blotting were consistent with those of RT-PCR (Fig. 3E). These results indicated that miRNA-200c-3p can inhibit the expression of EMT-related genes and proteins in TNBC, and Loss of miRNA-200c-3p can promote EMT of TNBC.

### Up-regulation of CRKL promotes the malignant phenotype of TNBC

**Fig. 4:**
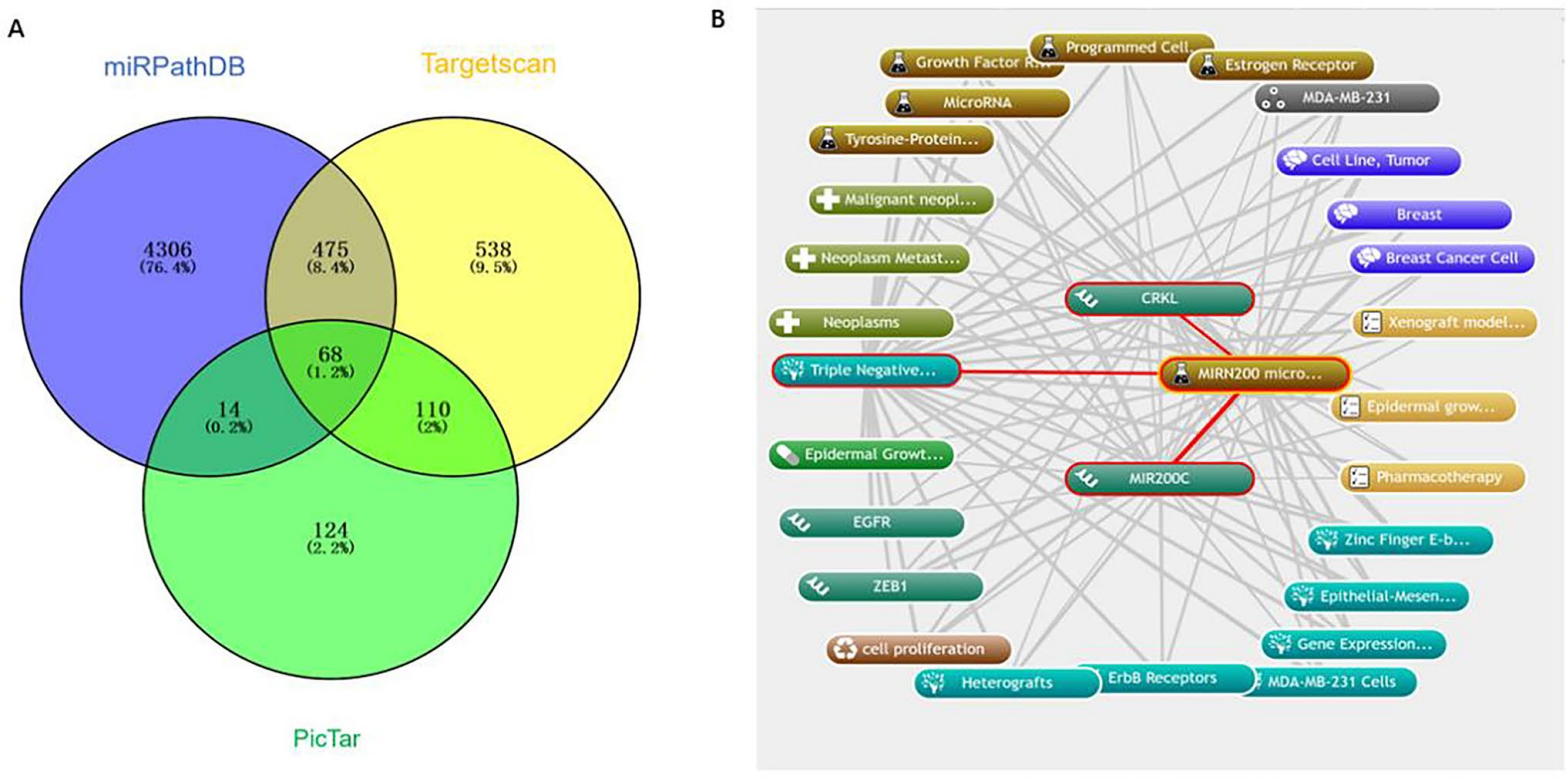
The potential targets of miRNA-200c-3p were searched in the database. **A** The intersection of potential targets of miRNA-200c-3p in miRPathDB, Targetscan and PicTar. **B** Bioinformatics and text mining performed by Coremine Medica

**Fig. 5:**
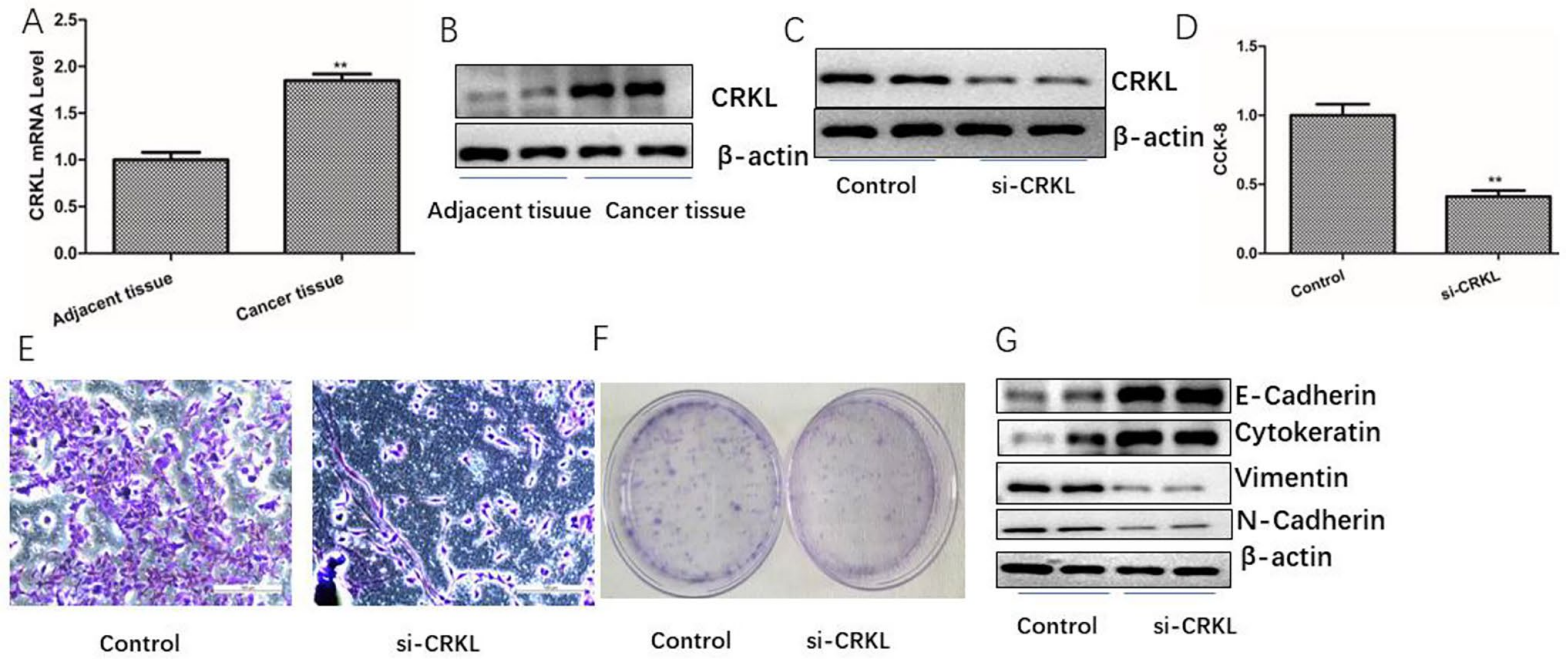
The expression of CRKL in tumor tissue and the effect of CRKL on the proliferation, invasion and EMT of TNBC. **A** Expression of CRKL mRNA in TNBC tissue. **B** Expression of CRKL protein in TNBC tissue. **C** The protein expression after CRKL knockdown was detected by western blot. **D** The changes of cell viability after CRKL knockdown were detected by CCK-8. **E** Transwell assay to verify the effect of si-CRKL on TNBC cells invasion. **F** Effect of CRKL knockdown on cell clonal formation. **G** The EMT-associated proteins expression after CRKL knockdown were detected by western blot

CRKL has been proved to be an oncogene, which plays a role in promoting tumor invasion and metastasis in various tumors involved in immune escape and EMT. In this study, the potential targets of miRNA-200c-3p were searched in miRPathDB, Targetscan and PicTar. And there are 68 potential targets at the intersection of the three databases (Fig. 4A). Then, bioinformatics and text mining performed by Coremine Medica, and found that among 68 potential targets, CRKL has the strongest correlation with EMT in TNBC (Fig. 4B). Further detection of CRKL expression in TNBC showed that CRKL was highly expressed in cancer tissue (Fig. 5A, B). To verify the effect of CRKL, we knock down CRKL and then western blot detected the knock down of CRKL (Fig. 5C). CCK-8 assay confirmed that CRKL knockdown could inhibit the activity of TNBC Cells (Fig. 5D). In the same time, Transwell assay and clonal formation showed that knockdown of CRKL can inhibit cells proliferation and invasion (Fig. 5E, F). Western blot assay showed that E-Cadherin and Cytokeratin protein expression were increased after CRKL was knocked down, and vimentin, N-Cadherin were decreased, indicated that CRKL promotes EMT of TNBC (Fig. 5G).

### miRNA-200c-3p Inhibit the expression of CRKL through by directly combining CRKL gene

**Fig. 6:**
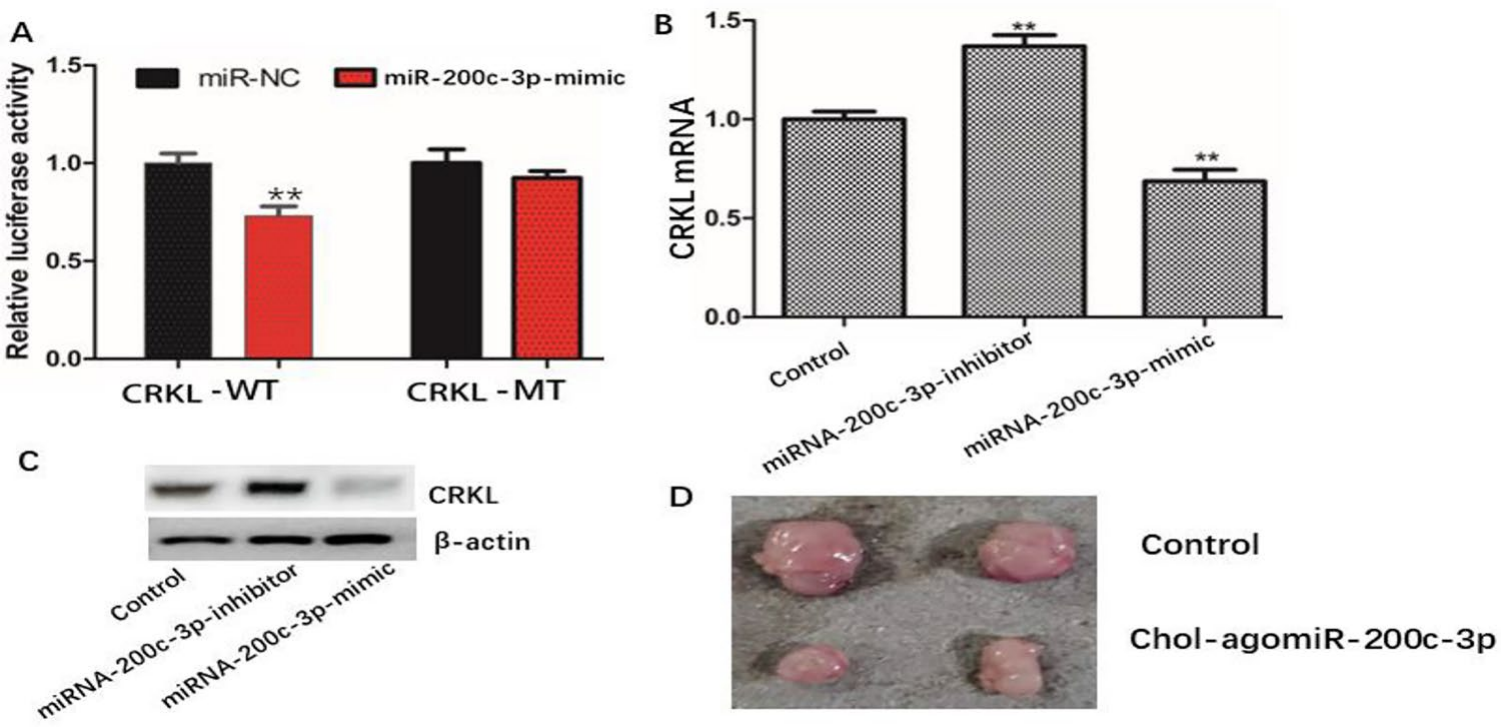
The effect of miRNA-200c-3p on CRKL and In vivo miRNA-200c-3p can inhibit tumor growth. **A** Luciferase reporter assay detected the effect of miRNA-200c-3p on CRKL. **B** RT-PCR detected the effect of miRNA-200c-3p on CRKL mRNA level. **C** Western blot detected the effect of miRNA-200c-3p on CRKL protein level. **D** The effect of miRNA-200c-3p on tumor was detected by subcutaneous grafting in nude mice. The values are presented as the means (SD), **p < 0.01 versus control group. All experiments were repeated three times

**Fig. 7:**
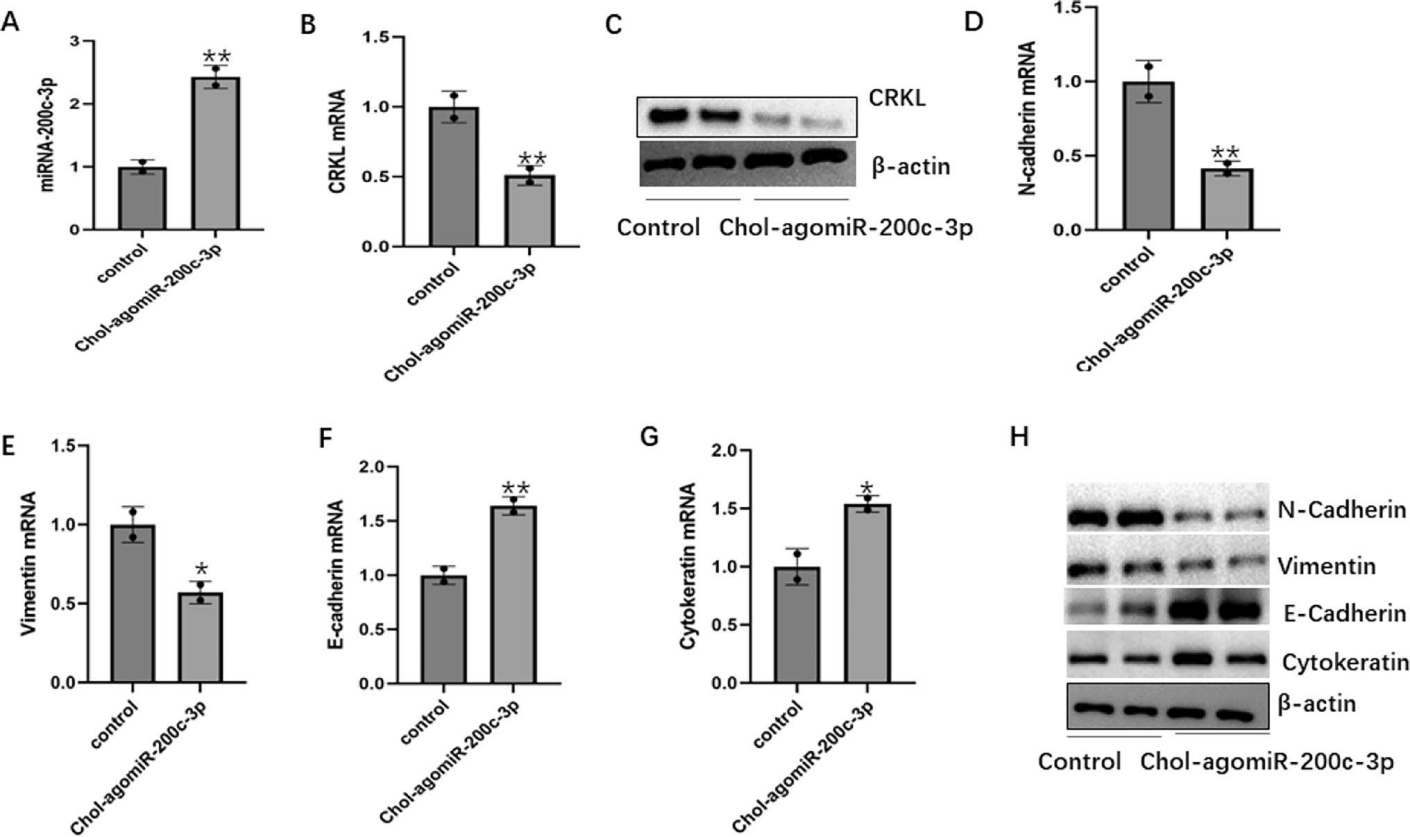
The effect of miRNA-200c-3p on CRKL and the effect of miRNA-200c-3p on EMT in tumors of nude mice. **A** Expression of miRNA-200c-3p mRNA. **B** Expression of CRKL mRNA. **C** Western blot detected the effect of miRNA-200c-3p on CRKL protein level. **D** Expression of N-cadherin mRNA. **E** Expression of Vimentin mRNA. **F** Expression of E-cadherin mRNA. **G** Expression of Cytokeratin mRNA. **H** The EMT-associated proteins were detected by western blot. The values are presented as the means (SD), **p < 0.01, *p < 0.05 versus control group. All experiments were repeated three times

Luciferase reporter assay detected that miRNA-200c-3p inhibit the expression of CRKL through by directly combining CRKL gene. RT-PCR and Western blot analysis showed that miRNA-200c-3p mimic decreased the expression of CRKL, and miRNA-200c-3p-inhibitor increased the expression of CRKL (Fig. 6A–C). These results indicated that miRNA-200c-3p inhibits the expression of EMT-related genes and proteins may through directly combining CRKL gene, inhibiting the malignant phenotype of TNBC. The tumor formation experiment in nude mice further confirmed that miRNA-200c-3p could inhibit the malignant phenotype of tumor cells (Fig. 6D). In the same time, the effect of miRNA-200c-3p on CRKL and the effect of miRNA-200c-3p on EMT in tumors of nude mice were also verified. RT-PCR showed that miRNA-200c-3p increased (Fig. 7A). RT-PCR and western blotting showed that CRKL level decreased (Fig. 7B, C). Western blotting and RT-PCR were performed to measure the expressions of EMT-related genes and proteins. We found that the expression of vimentin and N-cadherin decreased (Fig. 7D, E), E-cadherin, cytokeratin increased (Fig. 7F, G) and the results of western blotting were consistent with RT-PCR (Fig. 7H). These results indicated that miRNA-200c-3p can inhibit the expression of EMT-related genes and proteins in tumor, may by inhibiting CRKL expression.

## Discussion

TNBC accounts for 10–15% of all breast cancer styles [1, 2]. TNBC is easy to invade and metastasize, leading to poor prognosis and high mortality in patients. While the great improvement has been made towards treating and preventing TNBC with different methods such as chemotherapy, radiation therapy, and surgical operations but metastasis is still remaining the challenge due to the limits of effective treatment and early detection. The breast cancer likely to form metastasis in bone, lung, and liver. Therefore, it is very necessary to search for its invasion and metastasis mechanisms in TNBCs, and exploring intervention measures to improve the prognosis of TNBC is imperative.

The EMT is an important foundational process for tissue repair and organ development, and its abnormal activation has been proven to be a key step in tumor progression, distant metastasis, and drug resistance [24, 25]. In tumors, EMT is associated with the tumour stemness, invasion, metastasis and resistance to treatment [26–30], and which is a key step in TNBC cell metastasis. The occurrence of invasion and metastasis involves a variety of genes and signal transduction pathways, among which the roles of miRNAs is receiving increasing attention at home and abroad [31].

In recent years, miRNA as an effective biomarker or potential therapeutic target has received more and more attention. More and more studies have displayed that miRNA closely related to EMT affect the prognosis of tumors that through complex mechanisms. At present, a large number of research have shown that the miRNA plays a key role in pathogenesis, development, invasion and metastasis of breast cancer [32–34]. It has also been confirmed that the miR-200 family members can inhibit EMT by directly target the transcriptional regulators ZEBl and ZEB2 [10–12, 35]. However, whether EMT can be regulated by miRNA-200c-3p through other means has not been fully elucidated.

In this study, we found that miRNA-200c-3p lower expression and EMT activated abnormally in TNBC than matched tumor-adjacent tissues. We then tested it in the cell line and found that the results were consistent with those in the tissue. These results suggest that miRNA-200c-3p may play a role as a tumor suppressor in the development of TNBC, which will provide an important theoretical basis for small-molecule drug targeted therapy of breast cancer and the formation of a new pathway for clinical practice.

In order to determine the role of miRNA-200c-3p in TNBC and the effect of it on EMT, we conducted follow-up experiments to verify its function in TNBC. CCK-8 and transwell assay showed that miRNA-200c-3p-mimic significantly inhibited cell proliferation and cell invasion ability. To determine whether miRNA-200c-3p can inhibit EMT in TNBC, miRNA-200c-3p-mimic was exposed to TNBC cell lines, RT-PCT and western blot assay were performed to detect he expression of EMT-related genes and proteins in TNBC, displaying that miRNA-200c-3p-mimic Significantly upregulated the genes and proteins of E-cadherin and cytokeratin, and down-regulated the genes and proteins of vimentin and N-cadherin. Subcutaneous tumor transplantation in nude mice also confirmed that c can inhibit tumor growth and invasion. RT-PCT and western blot assay were performed to detect he expression of EMT-related genes and proteins in tumor of nude mice, displaying that miRNA-200c-3p. Significantly upregulated the genes and proteins of E-cadherin and cytokeratin, and down-regulated the genes and proteins of vimentin and N-cadherin. Which indicated that miRNA-200c-3p could inhibit EMT.

However, the site of action of miRNA-200c-3p still needs to be determined. To predict the target genes of miRNA-200c-3p, miRPathDB, Targetscan and PicTar database were used to identify targets. The target genes of the three databases were intersected, and there were 68 potential targets in total. Furthermore, bioinformatics and text mining performed by Coremine Medical confirmed that CRKL was significantly associated with EMT and malignant phenotype. To further confirm the effect of CRKL on TNBC, the CRKL gene was knocked down. And we found that knockdown of CRKL gene can inhibit the proliferation, invasion and EMT of TNBC. In order to verify the regulatory effect of miRNA-200c-3p on CRKL, the miRNA-200c-3p was overexpressed and interfered. Then, western blot was performed to detected CRKL protein expression level, and found that miRNA-200c-3p-mimic decreased CRKL protein expression level, as the same time miRNA-inhibitor promoted the expression of CRKL protein level. Luciferase reporter assay shows that miRNA-200c-3p can bind directly to CRKL mRNA, and then decreased CRKL protein expression level. Hence, we determined that miR-200c-3p can Inhibit the expression of CRKL through directly combining CRKL gene, and inhibit EMT, proliferation and invasion of TNBC.

## Conclusion

These results indicate that the loss of miRNA-200c-3p activates EMT by activating CRKL, and then promoting invasion and metastasis of TNBC.

## Data Availability

The data used to support the findings of this study are included within the article.
